# Facile and Controllable Fabrication of Protein-Only Nanoparticles through Photo-Induced Crosslinking of Albumin and Their Application as DOX Carriers

**DOI:** 10.3390/nano9050797

**Published:** 2019-05-24

**Authors:** Xiangyu Long, Jun Ren, Chao Zhang, Fangling Ji, Lingyun Jia

**Affiliations:** Liaoning Key Laboratory of Molecular Recognition and imaging, School of Bioengineering, Dalian University of Technology, No.2 Linggong Road, Dalian 116023, China; longxiangyu@mail.dlut.edu.cn (X.L.); iqiqi@mail.dlut.edu.cn (C.Z.); fanglingji@dlut.edu.cn (F.J.); lyjia@dlut.edu.cn (L.J.)

**Keywords:** protein-only nanoparticles, drug delivery, BSA, photo-induced crosslinking, biomedical application

## Abstract

Protein-based nanoparticles, as an alternative to conventional polymer-based nanoparticles, offer great advantages in biomedical applications owing to their functional and biocompatible characteristics. However, the route of fabrication towards protein-based nanoparticles faces substantial challenges, including limitations in size control and unavoidable usage of toxic crosslinkers or organic solvents, which may raise safety concerns related to products and their degradation components. In the present study, a photo-induced crosslinking approach was developed to prepare stable, size-controlled protein-only nanoparticles. The facile one-step reaction irradiated by visible light enables the formation of monodispersed bovine serum albumin nanoparticles (BSA NPs) within several minutes through a tyrosine photo-redox reaction, requiring no cross-linking agents. The size of the BSA NPs could be precisely manipulated (from 20 to 100 nm) by controlling the duration time of illumination. The resultant BSA NPs exhibited spherical morphology, and the α-helix structure in BSA was preserved. Further study demonstrated that the 35 nm doxorubicin (DOX)-loaded BSA NPs achieved a drug loading content of 6.3%, encapsulation efficiency of 70.7%, and a controlled release profile with responsivity to both pH and reducing conditions. Importantly, the in vitro drug delivery experiment demonstrated efficient cellular internalizations of the DOX-loaded BSA NPs and inhibitory activities on MCF-7 and HeLa cells. This method shows the promise of being a platform for the green synthesis of protein-only nanoparticles for biomedical applications.

## 1. Introduction

Therapeutic nanoparticles (NPs) are attracting significant attention for their great promise in numerous medical applications, including drug delivery, imaging, vaccine formulations, and biodetection [1,2]. Among these applications, tremendous efforts have been made in improving cancer treatment, in which nanosized carriers can exhibit attractive features for target delivery, prolonging circulation time, and decreasing the systemic toxicity of chemotherapy agents [3]. During the last decade, a range of material platforms for drug delivery have been developed, including inorganic and polymer-based nanoparticles, micelles, liposomes, and protein nanoparticles [4].

The intrinsic biocompatibility of drug carriers is one of the significant considerations for drug delivery systems. Among the numerous kinds of nanoplatforms, protein nanoparticles possess several advantages compared to other materials, including good biocompatibility, easy accessibility, and low toxicity of degradation products [5,6]. Albumin-based nanoparticles, which have already realized packing and delivering a variety of chemotherapy agents [7,8,9], have enormous commercial values. Albumin is the most abundant native protein in the peripheral circulation with intrinsic biocompatibility and high solubility in blood. Additionally, albumin has natural binding sites for nonpolar molecules because it is amphiphilic, which makes it especially suitable for packing hydrophobic drugs [10,11,12].

In the past decades, in regard to the fabrication of albumin-based nanoparticles, several methods have been developed including desolvation, emulsification, thermal gelation, and spray drying [13,14,15,16,17]. However, the current particle fabrication processes remain unsatisfactory, mainly because of poor control of size and safety concerns associated with the usage of toxic crosslinkers or organic solvents [9,18,19,20]. In a typical emulsion method, albumin nanoparticles are synthesized generally by the continuous addition of ethanol into an albumin solution followed by crosslinking with glutaraldehyde. Harsh conditions would lead the denaturation and unpredictable biocompatibility of albumin; however, in the solution without organic solvents, random crosslinking of albumin molecules may lead to aggregation and unsatisfactory size control, especially for sub-100 nm particles. Besides, other ways such as heating aggregation may produce hydrophobicity-driven protein assembly with weak stability [17]. Hence, although various methods have been reported, a novel size-controlled and facile fabrication of protein nanoparticles remains in urgent need.

Photochemical processes have been reported in many studies to facilitate efficacious catalytic processes with mild conditions, and photo-induced crosslinking between biomacromolecules has also been developed [21]. Fancy reported that tyrosine residues of proteins can be cross-linked under a visible light-irradiated conditions, and ruthenium (Ru(II)) played an important role as a photoredox initiator [22]. This photo-induced radical crosslinking reaction enables efficient formation of a multiprotein assembly. The photoredox system has been applied to analyze the aggregation of the amyloid β-protein (Aβ), and it has also been widely used to crosslink tyrosine-rich proteins such as gelatin and fibrinogen into hydrogels for tissue engineering materials [23,24,25,26]. For these applications, the photochemical method is featured by high efficiency and mild reaction conditions. The reaction was even mild enough for patterning adhesion of mammalian cells [27]. Our previous study also proved that such a Ru(II)-catalyzed photochemical process can realize highly efficient crosslinking between bioactive proteins and a range of phenol-functionalized material platforms, the reaction condition allowing lipase, Staphylococcus aureus protein A, and streptavidin to maintain their bioactivity [28].

**Scheme 1:**
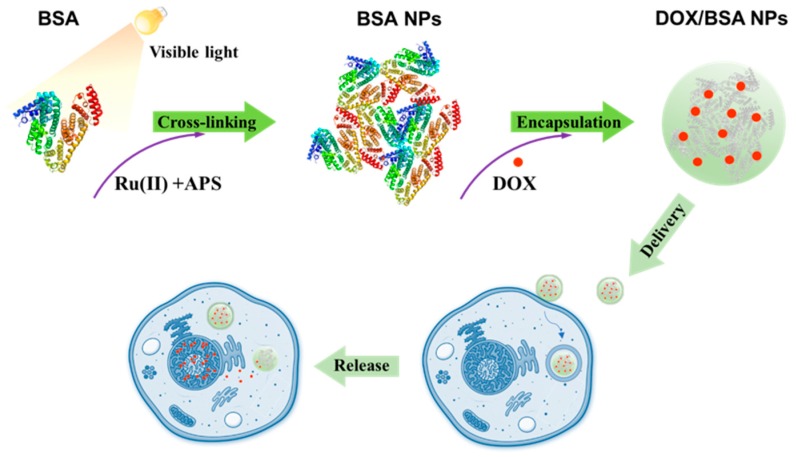
Illustration of the fabrication of drug-loaded bovine serum albumin nanoparticles (BSA NPs) and their application as a doxorubicin (DOX) vehicle.

In this study, we proposed a photochemical crosslinking approach for fabricating crosslinker-free protein-only nanoparticles. Bovine serum albumin (BSA) was chosen as the model protein. We sought to maintain the natural structure of BSA molecules during the assembly and crosslinking process so that their natural binding pockets could be kept for packing hydrophobic drugs. The BSA NPs were optimized for drug encapsulation toward doxorubicin (DOX), an anticancer chemotherapeutic drug, and the cellular uptake and cytotoxicity were studied in comparison with free DOX (Scheme 1).

## 2. Materials and Methods

### 2.1. Materials and Reagents

Bovine serum albumin (BSA), doxorubicin hydrochloride (DOX·HCl), and ammonium persulphate (APS) were purchased from Solarbio Technology Co., Ltd. (Beijing, China). Tris-bipyridyl ruthenium (II) chloride (Ru(bpy)_3_Cl_2_) was obtained from Tokyo Chemical Industry Co., Ltd. (Shanghai, China). Dithiothreitol (DTT) was purchased from Aladdin Industrial Corporation (Shanghai, China). Fluorescein isothiocyanate (FITC) was obtained from J&K Scientific (Beijing, China). All chemicals and reagents were of analytical grade and were used as purchased. An ultrapure water purification system (Millipore, Billerica, MA, USA) was used to obtain Milli-Q^®^ ultrapure water that reached a resistivity of 18.2 MΩ·cm, and Milli-Q® ultrapure water was used in all experiments.

Human breast cancer cell line (MCF-7) and cervical cancer cell line (HeLa) were purchased from Shanghai Institute for Biological Sciences, Chinese Academy of Science (Shanghai, China). Dulbecco’s modified Eagle’s medium (DMEM), penicillin-streptomycin solution (100×), fetal bovine serum (FBS), and 0.25% trypsin-EDTA (1×) solution for cell culture were purchased from Hyclone (Logan, UT, USA).

### 2.2. Methods

#### 2.2.1. Preparation of Bovine Serum Albumin Nanoparticles (BSA NPs)

Briefly, lyophilized BSA (3 mg) was dissolved in 300 μL phosphate buffer solution (PBS, 0.01M, pH 7.4), followed by the addition of 10 μL Ru(bpy)_3_Cl_2_ and 10 μL APS stock solutions to achieve a final concentration of 10 mg/mL BSA, 0.35 mM Ru(bpy)_3_Cl_2_, and 7 mM APS. Crosslinking of BSA was triggered by the illumination of visible light generated from a 200 W incandescent lamp. The sizes of the BSA NPs were precisely controlled through the duration of illumination. Illumination times from 120 to 480 s were employed. DTT was added into the system (with the final concentration of 20 mM) to stop the reaction. The resultant samples were further ultrafiltered (molecular weight cutoff: 10 kDa) with phosphate buffer solution (PBS, pH 7.4) to remove the reaction reagents. The concentration of residual Ru(II) in purified BSA NPs was measured by an inductive coupled plasma (ICP) emission spectrometer (Optima 2000DV, PerkinElmer, Waltham, MA, USA). The residual rate (RR) of Ru(II) was calculated using the following equation:

RR = MN/MT × 100%,(1)

where MT is the total moles of Ru(II) fed, and MN represents the moles of Ru(II) in purified nanoparticles.

#### 2.2.2. Preparation of Doxorubicin (DOX)/BSA NPs

Different volumes of 10 mM DOX·HCl solutions were added into BSA NP solutions (4 mg/mL in double distilled water (ddH_2_O)) to reach mass ratios of 1:5, 1:10, and 1:20. The mixture was then adjusted to pH 8.0 and stirred in the dark for 2 h. Free DOX in the solution was separated from DOX-loaded BSA NPs (DOX/BSA NPs) by several rounds of ultrafiltration. The ultrafiltrate was collected, and the free DOX concentrations were determined by a spectrophotometer (SpectraMax M2e, Molecular Device, Sunnyvale, CA, USA) at 480 nm. The adsorption of DOX on the ultrafiltration membrane was normalized to a standard free doxorubicin solution at the same condition. Encapsulation efficiency (EE) and drug loading content (LC) were calculated using the following equations:

EE = (WT − WF)/WT × 100%,(2)

LC = (WT − WF)/WNP × 100%,(3)

where WT is the total weight of the drug fed, WF represents the weight of the nonencapsulated free drug, and WNP indicates the weight of nanoparticles. After lyophilization, DOX/BSA NPs were obtained for further use.

#### 2.2.3. Characterizations

Zeta potential, nanoparticle volume-weighted size distribution, and monomeric protein versions were determined using a dynamic light scattering (DLS) analyzer (Zetasizer Nano ZS90, Malvern Instruments Limited, Malvern, UK). The samples were diluted to an identical concentration of 0.5 mg/mL before measurement, and all determinations were conducted at a scattering angle of 90° and equilibrated to 25 °C. Data were produced from three independent experiments and presented as mean ± standard deviation (SD).

Transmission electron microscope (TEM) analysis was performed to visualize the morphology of nanoparticles. A JEM-2100 transmission electron microscopy (JEOL, Tokyo, Japan) was used for the analysis. Solutions of nanoparticles (0.5 mg/mL in ddH_2_O, 10 μL) were placed onto a glow-discharged and carbon-coated 200-mesh copper grid, and excess material was then removed. The samples were negatively stained with 2% phosphotungstic acid for 1 min and air-dried.

Conformational changes of the protein caused by photochemical crosslinking were analyzed by a circular dichroism (CD) spectrometer (MOS-500, Biologic, Seyssinet Pariset, France) at room temperature. BSA samples of blank nanoparticles, drug-loaded nanoparticles, and monomeric protein versions were prepared in phosphate buffer (10 mM) and injected into a 1.0 mm path length quartz cuvette. The spectrum scope was from 190 to 250 nm with a scanning rate of 60 nm per minute. Data of every single sample were obtained and averaged over three scanning times.

Fourier transform infrared spectroscopy (FTIR) analysis was operated to determine the change of the chemical bonds of BSA after crosslinking. A Nicolet iS5 spectrometer (Thermo Fisher Scientific, Waltham, MA, USA) equipped with an iD7 ATR accessory was used for test. Briefly, 1–2 mg powder samples of lyophilized native BSA and BSA NPs were flattened and placed under the probe, respectively. Both of the spectra were collected in the range of 500–4000 cm^−1^. Data of every sample were obtained and averaged over ten scanning times.

#### 2.2.4. In Vitro Drug Release of DOX/BSA NPs

The in vitro release profile of DOX from DOX/BSA NPs was monitored using a dialysis method. DOX/BSA NPs solution (2 mL) in a dialysis bag with molecular weight cut off (MWCO) of 14 kDa was dialyzed against 100 mL release buffer at 37 °C. Release buffer (1 mL) was periodically removed from the system with the addition of an equivalent volume of fresh buffer. The system was read at 480 nm to determine the concentration of doxorubicin in the release buffer. Data were collected and averaged from three independent experiments.

#### 2.2.5. Cellular Internalization Assay

BSA NPs were labeled with FITC to facilitate tracker analysis. Briefly, a mixture of 300 μL of FITC solution (1 mg/mL in anhydrous DMSO) and 3 mL of BSA NPs solution (10 mg/mL in 0.1 M carbonate buffer, pH 9.0) were stirred at room temperature for 2 h, which was followed by another 24 h of reaction at 4 °C in the dark. The reaction mixture was then purified via ultrafiltration (MWCO 10 kDa) with carbonate buffer (pH 9.0). FITC-labeled BSA NPs were used to prepare DOX/FITC-BSA NPs as mentioned above. After lyophilization, DOX/FITC-BSA NPs were obtained for further use.

MCF-7 cells were seeded into DMEM medium containing 10% FBS with a density of 1 × 10^5^ cells per glass bottom dish at 37 °C and 5% CO_2_. After 24 h, DOX/BSA NPs were added reaching a final DOX concentration of 10 μg/mL and co-incubated for either 4 or 8 h. After removal of the culture media, MCF-7 cells were washed with PBS three times to remove NPs that were not internalized. The nuclei were subsequently stained with 10 μL (1 mg/mL) of Hoechst 33342 and incubated for another 15 min. Finally, the cells were washed three times using PBS and resuspended in 1 mL DMEM. The intracellular distribution of DOX was confirmed using FV1000 confocal laser scanning microscopy (Olympus, Tokyo, Japan).

#### 2.2.6. Cytotoxicity Assay

The cytotoxicity of DOX/BSA NPs and free DOX against MCF-7 and HeLa cells were evaluated by using a cell counting kit-8 (CCK-8) assay. DMEM medium with a supplement of 10% FBS was used for the maintenance of the cells. The MCF-7 and HeLa cells in the exponential growth phase were cultured in 96-well plates at a density of ~1 × 10^4^ cells per well in the condition of 37 °C and 5% CO_2_. After incubation for 24 h, cell culture media was replaced by fresh media containing serially diluted concentrations, ranging from 1.0 mg/mL to 1.0 ng/mL, of free DOX or DOX/BSA NPs (equivalent concentration to free DOX) followed by another 48 h incubation. Then, the media was discarded, and the cells were washed. Finally, the cells were incubated for another 2 h with the addition of 100 μL of fresh medium supplemented with 10 μL of CCK-8 solution, and the absorbance was measured by using a microplate reader (Thermo Fisher Scientific, USA) at 450 nm. Each test was performed in triplicate and averaged, and cell viability was expressed as a percentage of the control culture value.

## 3. Results and Discussion

### 3.1. Preparation and Characterization of BSA NPs

**Figure 1:**
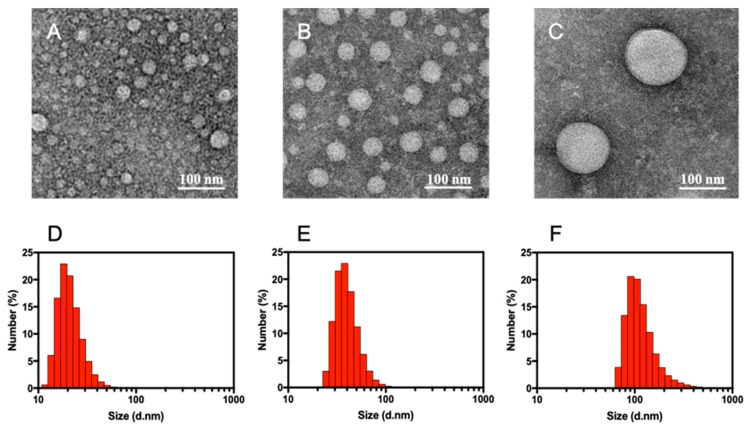
Transmission electron microscope (TEM) images (**A**–**C**) and hydrodynamic diameters determined with dynamic light scattering (DLS) of BSA NPs (**D**–**F**) at 20, 40, and 100 nm. The scale bar represents 100 nm.

BSA NPs were prepared at physiological conditions by illuminating the BSA solution that contained Ru(II) and APS with a 200 W incandescent lamp. As shown in Table 1, photo-redox crosslinking between BSA molecules could be completed within less than 10 min, and the size of protein nanoparticles could be manipulated by controlling the duration time of illumination. DLS measurements of BSA NPs revealed a size range of 20–100 nm dependent on the reaction time (Table 1). According to TEM micrographs (Figure 1), the resultant BSA NPs exhibited a spherical shape with a good monodispersion. This may be because BSA molecules could still maintain their natural globular shape when they assembled into multimolecular nanoparticles. In contrast, irregularly shaped protein nanoparticles were usually obtained with some methods using denatured protein as building blocks, for example, through heating treatment or emulsification methods, in which proteins lost their structures and aggregated randomly [14,17,29].

The ratio of initiator molecule to protein molecule can also influence the process of nanoparticle formation. A higher ratio of Ru(II) can accelerate the crosslinking reaction by generating a higher concentration of free radicals. As shown in Table 1, when the Ru(II)/BSA ratio increased from 0.5 to 2, a much shorter time was needed to achieve a similar degree of reaction. With the higher dosage of Ru(II), an illumination period of 280 s was enough to generate BSA NPs of about 100 nm in average size, while 480 s was needed to achieve a similar particle size when a lower dosage of Ru(II) was used. Note that for both conditions, it was hard to prepare BSA NPs beyond 100 nm with satisfactory size distributions by further prolonging the reaction time. Phase transformation could occur, and the bulk of the protein aggregates tended to form protein hydrogels. Actually, sub-100 nm BSA NPs are desirable for drug delivery applications because particles larger than 100 nm would be quickly cleaned from the circulation by phagocytes of the reticuloendothelial system [30].

Ru(II) residue in the obtained BSA NPs, which may raise safety concerns, was determined after the BSA NPs were fully washed. As shown in Table 2, nearly 90% of the Ru(II) fed to the reaction system could be removed, while the residual Ru(II) in BSA NPs was found to be 0.009% and 0.046% (w/w) for the above two reaction systems, respectively. Previous study reported that Ru(II) had no significant toxicity to human chondrocytes even at millimolar concentrations [26]. By comparison, the residual amount of Ru(II) in BSA NPs was much lower than the reported level, so safety concerns related to Ru(II) residue were not significant.

**Figure 2:**
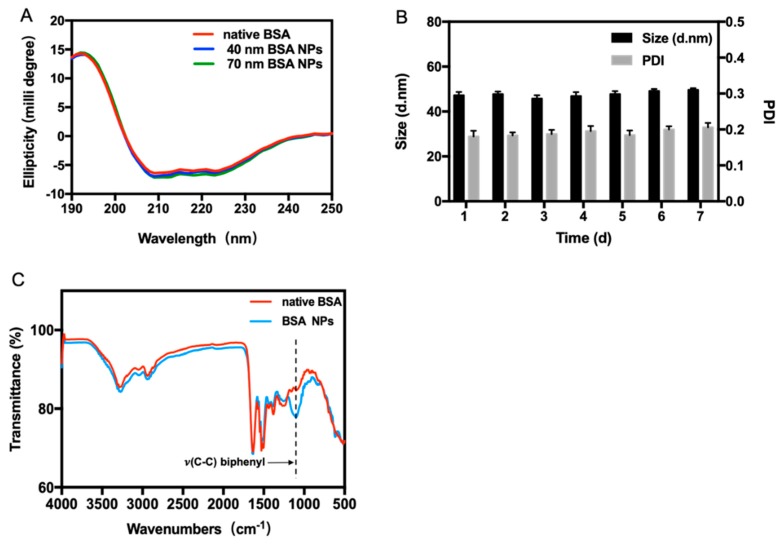
Secondary structure, stability property, and Fourier transform infrared spectroscopy (FTIR) characterization of BSA NPs. (**A**) Far-UV circular dichroism (CD) spectra of native BSA and BSA NPs of different sizes. Representative spectra are the average of three consecutive scans and smoothed with a Savitzy−Golay least-squares fit. (**B**) Change of the particle size of BSA NPs over a period of 7 d according to DLS measurements. The sample was stored in PBS (pH 7.4, 150 mM NaCl) at 4 °C. (**C**) FTIR spectra of native BSA and BSA NPs.

Maintenance of the native structure is of great significance for albumin-based anticancer chemotherapeutic drug delivery systems. Native albumin can utilize albumin receptor (gp60) and albumin-binding protein called secreted protein acidic and rich in cysteine (SPARC), which is an extracellular matrix glycoprotein overexpressed in various cancers, to enhance uptake of drugs in solid tumors [9]. However, most current approaches established to prepare albumin-based nanoparticles cannot avoid the denaturation of albumin molecules. To determine the influence of tyrosine-based crosslinking on the structural integrity of BSA, a CD analysis was conducted to detect conformational alterations in the secondary structure (Figure 2A). The featured peaks at 208 and 222 nm were typical marks of an α-helix structure, the content of which reflected conformational alteration of BSA. The results of Figure 2A showed that the CD spectra of BSA NPs of both 40 and 70 nm were almost identical to that of native BSA molecules, manifesting the well-maintained predomination of an α-helix structure in BSA [31].

The stability of BSA NPs was further investigated by testing possible aggregation during storage. No precipitation was observed in the sample solution after being stored at 4 °C for 1 week, and no significant changes in particle size and polydispersity index (PDI) were found by DLS analysis (Figure 2B), which indicated a good resistance to spontaneous aggregation. There might be two factors contributing to the good stability of BSA NPs prepared in this study. First, BSA molecules were crosslinked mainly through tyrosine residues on the protein chain, and the reaction would not alter their zeta potential. Second, the crosslinked BSA molecules retained their spatial structures without exposing inner hydrophobic regions. Therefore, in spite of being crosslinked together, BSA molecules involved in the nanoparticles retained their intact surface properties to a large extent. Such structural integrity of each individual protein also guaranteed the overall stability of the BSA NPs.

FTIR was performed to detect the resultant chemical bonds after photo-induced crosslinking. Compared to the infrared spectrum of native BSA, a strong absorption peak at 1138 cm^−1^ appeared in the BSA NPs spectrum, which belonged to the biphenyl C–C vibration peak (Figure 2C). This confirmed that BSA NPs formed mainly through crosslinking between tyrosine residues, generating dityrosine to link individual monomeric proteins together. This type of chemical crosslinking can guarantee a much stronger binding between monomers compared to physical interactions, and it could also contribute to the overall stability of BSA NPs.

### 3.2. Preparation and Characterization of DOX/BSA NPs

As a high-abundance protein in plasma, albumin biologically acts as a transporter, and it can bind to a wide range of drugs and nonpolar metabolites (like bilirubin) through noncovalent interactions. To explore the possible application of BSA NPs as a drug vehicle, DOX was used as a model chemotherapeutic drug.

BSA NPs of about 20 nm were used to load DOX by direct incubation at room temperature, and the amount of DOX was varied to optimize encapsulation performance. The resultant DOX/BSA NPs were characterized for their DOX loading content (LC), encapsulation efficiency (EE), particle size, and zeta potential. The data of Table 2 showed that with a mass ratio of DOX to BSA NPs increasing from 5% to 20%, LC increased from 3.8% to 10.6%, while EE decreased from 75.5% to 52.8%. The data reflected a remarkable loading capacity of the BSA NPs toward DOX, since LC values of 2–6% were normally reported for other BSA-based encapsulation systems [32].

Table 3 presents the variation of size and zeta potential of the DOX/BSA NPs as a function of LC. The sizes of the resultant nanoparticles showed a slight increase with the increase in LC, while the zeta potential increased slightly at the same time. The increased size of DOX/BSA NPs can be attributed to the further aggregation of BSA molecules in the presence of DOX molecules. Since BSA has an isoelectric point (pI) of 4.7, it can be negatively charged under weakly alkaline conditions. With the positively charged DOX added, the intermolecular attractions between DOX and BSA could lead to the decrease of negative surface charges of BSA, and then larger nanoparticles may gradually form. Therefore, the results of zeta potential coincided with particle size. A similar phenomenon has been reported in other studies [10,33].

**Figure 3:**
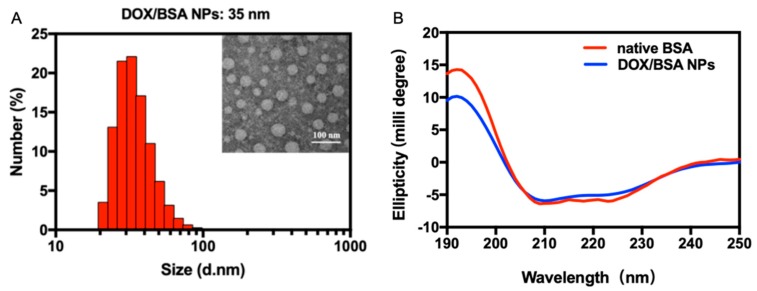
Characterization of secondary structure and particle size of DOX/BSA NPs. (**A**) TEM images of DOX/BSA NPs and the particle size of DOX/BSA NPs measured by DLS. (**B**) Far-UV CD spectra of DOX/BSA NPs. Representative spectra are the average of three consecutive scans and smoothed with a Savitzy−Golay least-squares fit.

From the above results, it can be confirmed that DOX molecules could be loaded into BSA NPs. Over and above electrostatic interaction, hydrophobic interaction should be principally accountable for DOX encapsulation into BSA NPs. Taking both LC and EE into consideration, DOX/BSA NPs with the LC of 6.3% were chosen for further investigation. TEM images showed that DOX/BSA NPs were also spherical with a relatively uniform diameter (Figure 3A). The average particle size was 35.3 ± 3.8 nm in diameter according to DLS measurements, which was in agreement with the result observed from TEM. As shown in Figure 3B, the CD spectra of DOX/BSA NPs nm were also similar to that of native BSA molecules, indicating that the process of encapsulating DOX did not have a significant impact on the secondary structure of BSA, and its α-helix structure can be well maintained.

### 3.3. In Vitro Drug Release

**Figure 4:**
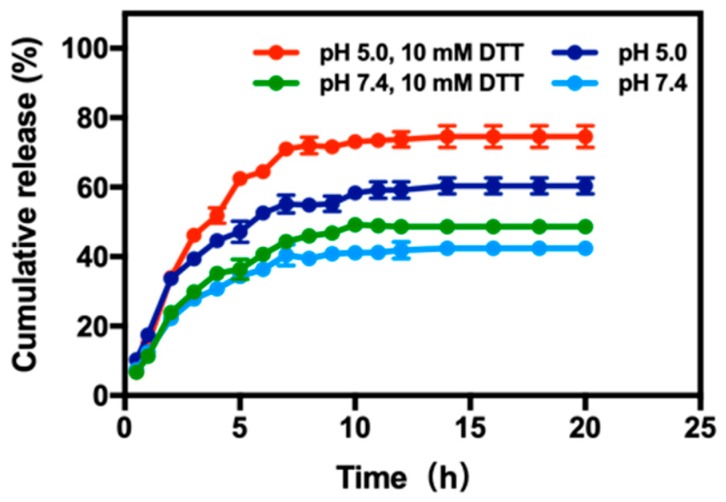
In vitro DOX release profiles from DOX/BSA NPs under different conditions at 37 °C.

DOX release behavior was measured in different buffers as exhibited in Figure 4. In vitro release of DOX from DOX/BSA NPs was studied at physiological pH and acidic pH, respectively. In addition, the influence of 10 mM DTT also mimicked intracellular reductive conditions [34].

No burst release was observed. The cumulative drug release ratio increased in the first 8 h and maintained a stable growth in the next 10 h. DOX releases observed from DOX/BSA NPs in physiological and weak acidic pH over a period of 20 h were 40% and 60%, respectively. The drug release rate evidently became faster at pH 5.0, which was attributed to the weaker interaction between DOX and BSA. The hydrophilicity of DOX increased, on account of low pH, and DOX encapsulated by BSA may escape from the interior hydrophobic region of BSA more easily.

Reductive conditions can also have a conspicuous impact on drug release behaviors. In the presence of 10 mM DTT in PBS, the cumulative DOX releases from DOX/BSA NPs at pH 7.4 and pH 5.0 were 47% and 75%, respectively, in 20 h. The effect of DTT can be explained by its potential impact on the conformation of BSA. BSA contains 17 disulfide bonds, which play a key role in maintaining the natural conformation of BSA. Reductive conditions can impair the structural stability of the protein to a certain degree by reducing these disulfide bonds, leading to the loss of some natural binding sites for hydrophobic drugs. Therefore, increased conformational flexibility of BSA NPs in reductive conditions may boost the release of encapsulated DOX.

The maximum cumulative drug releases in acidic and reductive conditions were about 87.5% higher than those in physiological conditions. The results showed that the DOX/BSA NPs had a positive response to weak acidic and reductive conditions. Under physiological pH conditions, a slow drug release rate might be helpful to reduce the toxicity of anticancer drugs during circulation; the controlled release of drugs can be ensured at tumor sites or inside cells, where pH values are lower than serum. This performance is beneficial for an anticancer drug delivery vehicle to realize tumor-targeted functions.

### 3.4. Cellular Internalization of DOX/BSA NPs

**Figure 5:**
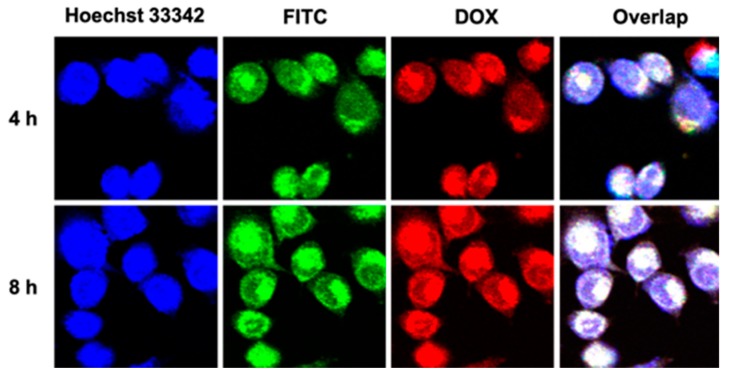
Confocal images of MCF-7 cells after being treated by DOX/BSA NPs for 4 and 8 h. The blue fluorescence showed cell nuclei were stained with Hoechst 33342. The green fluorescence represents FITC, which was labeled on BSA.

To further study cellular uptake behavior, confocal microscopy was operated to observe the cellular internalization of DOX/BSA NPs by MCF-7 cells. As shown in Figure 5, not only green fluoresce (FITC-BSA) but also red fluoresce (DOX) can be detected in the cytoplasm and cell nuclei after 4 h of incubation, confirming cell internalization of the DOX/BSA NPs [33]. By prolonging the incubating time to 8 h, the intensity of both green and red fluoresce became higher, demonstrating MCF-7 cells enhanced the uptake capacity of DOX/BSA NPs.

### 3.5. In Vitro Cytotoxicity Assays

**Figure 6:**
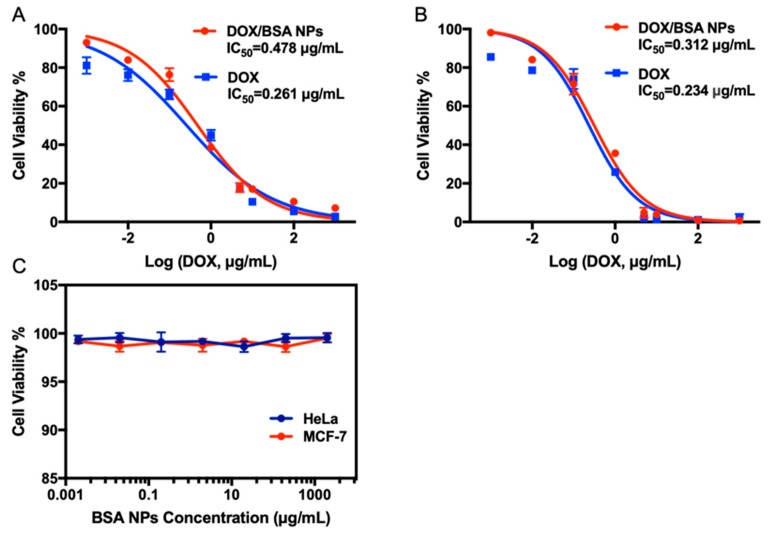
Cytotoxicity of empty BSA NPs and DOX/BSA NPs against cancer cells. The cell viability of HeLa cells (**A**) and MCF-7 cells (**B**) after 48 h incubation with free DOX and DOX/BSA NPs. (**C**) HeLa and MCF-7 cell viability after incubated (48 h) with BSA NPs. Data are presented as mean ± SD, *n* = 6.

The in vitro cytotoxicity of BSA NPs in HeLa and MCF-7 cells was assessed by a CCK-8 assay (Figure 6). Results confirmed that the cytotoxicity of pure blank BSA NPs without embedding DOX was inappreciable in both HeLa and MCF-7 cells even at a high concentration (Figure 6C). Tumor cell inhibition efficiency of DOX/BSA NPs was further characterized by measuring the half maximal inhibitory concentration (IC_50_) to HeLa and MCF-7 cells, respectively. Figure 6A,B indicated that both DOX/BSA NPs and free DOX exhibited dose-dependent cytotoxicity, and the inhibitory effect of DOX/BSA NPs was found parallel with that of free DOX against both types of cells. For both Hela cells and MCF-7 cells, DOX/BSA NPs showed a bit lower cytotoxicity compared to free DOX, which indicated that DOX/BSA NPs had a good biocompatibility. The above results proved that DOX/ BSA NPs could achieve intracellular delivery and the release of DOX to induce effective cytotoxicity against cancer cells.

## 4. Conclusions

A novel approach was established to construct protein-only nanoparticles for drug delivery applications. The facile, one-step photo-crosslinking method enabled formation of stable and monodispersed protein nanoparticles within several minutes through a tyrosine photo-redox reaction, and no cross-linking agents were needed. Different from previously reported methods, protein molecules did not need to be exposed to heat or organic reagent-mediated denaturation conditions, and they could maintain their natural structure to a large extent. The fabrication procedure required no organic solvents and surfactants, making the synthesis of BSA NPs a completely green process. With a model system, we proved that the resultant BSA NPs had a good cytocompatibility and could effectively deliver DOX in a controlled manner. This method shows the promise of being a platform for fabrication of bioactive protein nanoparticles for biomedical applications.

## Figures and Tables

**Table 1:** Size control of BSA NPs through illumination time at different mole ratios of initiator to BSA. Data were collected from dynamic light scattering (DLS) measurements and presented as mean ± SD, *n* = 3.

**Ru(II):BSA = 1:0.5** **(mol:mol)**	**Time (s)**				
	**0**	**120**	**240**	**270**	**280**
Diameter (nm)	6.7 ± 0.4	29.5 ± 0.8	40.8 ± 1.5	73.0 ± 4.2	105.4 ± 3.5
Polydispersity index (PDI)	0.176 ± 0.004	0.189 ± 0.014	0.195 ± 0.012	0.212 ± 0.008	0.239 ± 0.016
**Ru(II):BSA = 1:2** **(mol:mol)**	**Time (s)**				
	**0**	**180**	**300**	**420**	**480**
Diameter (nm)	6.7 ± 0.3	29.4 ± 1.5	45.2 ± 3.3	64.4 ± 5.1	98.6 ± 7.4
Polydispersity index (PDI)	0.180 ± 0.004	0.185 ± 0.013	0.165 ± 0.024	0.228 ± 0.011	0.252 ± 0.005

**Table 2:** The statistics of the residual rate (RR) of Ru(II) and the concentration of Ru(II) in 30 nm BSA NPs at different mole ratios of initiator to BSA.

Ru(II):BSA (mol:mol)	RR	Concentration of Ru(II) (μm)	Concentration of BSA NPs (mg/mL)	Residual Ru(II) in BSA NPs (w/w)
1:0.5	11.4%	5.9	1.3	0.0046%
1:2	12.3%	6.4	5.8	0.009%

**Table 3:** Structural and drug loading properties of DOX/BSA NPs. Data are presented as mean ± SD, *n* = 3.

Drug/carrier (m/m)	Loading Content (LC) (%)	Encapsulation Efficiency (EE) (%)	Size (nm)	PDI	Zeta potential (mV)
1:5	10.6 ± 0.2	52.8 ± 2.4	40.4 ± 3.3	0.23 ± 0.03	−10.4 ± 1.4
1:10	6.3 ± 0.1	70.7 ± 1.2	35.3 ± 3.8	0.25 ± 0.09	−12.5 ± 0.6
1:20	3.8 ± 0.2	75.5 ± 2.6	32.1 ± 1.9	0.28 ± 0.07	−13.1 ± 1.1
